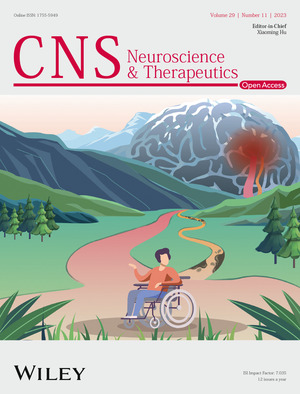# Front cover

**DOI:** 10.1111/cns.14505

**Published:** 2023-10-17

## Abstract

The cover image is based on the Original Article *Mediation effect of stroke recurrence in the association between post‐stroke interleukin‐6 and functional disability* by Hong‐Qiu Gu et al., https://doi.org/10.1111/cns.14289.